# Obese Rats Exhibit High Levels of Fat Necrosis and Isoprostanes in Taurocholate-Induced Acute Pancreatitis

**DOI:** 10.1371/journal.pone.0044383

**Published:** 2012-09-18

**Authors:** Javier Pereda, Salvador Pérez, Javier Escobar, Alessandro Arduini, Miguel Asensi, Gaetano Serviddio, Luis Sabater, Luis Aparisi, Juan Sastre

**Affiliations:** 1 Department of Physiology, School of Pharmacy, University of Valencia, Burjasot, Valencia, Spain; 2 Division of Neonatology, University Hospital Materno-Infantil La Fe, Valencia, Spain; 3 Department of Medical and Occupational Sciences, University of Foggia, Foggia, Italy; 4 Department of Surgery, University of Valencia, Universitary Clinic Hospital, Valencia, Spain; 5 Laboratory of Pancreatic Function, Universitary Clinic Hospital, Valencia, Spain; The Ohio State Unversity, United States of America

## Abstract

**Background:**

Obesity is a prognostic factor for severity in acute pancreatitis in humans. Our aim was to assess the role of oxidative stress and abdominal fat in the increased severity of acute pancreatitis in obese rats.

**Methodology:**

Taurocholate-induced acute pancreatitis was performed in lean and obese Zucker rats. Levels of reduced glutathione, oxidized glutathione, L-cysteine, cystine, and S-adenosylmethionine were measured in pancreas as well as the activities of serine/threonine protein phosphatases PP1 and PP2A and tyrosin phosphatases. Isoprostane, malondialdehyde, triglyceride, and free fatty acid levels and lipase activity were measured in plasma and ascites. Lipase activity was measured in white adipose tissue with and without necrosis and confirmed by western blotting.

**Findings:**

Under basal conditions obese rats exhibited lower reduced glutathione levels in pancreas and higher triglyceride and free fatty acid levels in plasma than lean rats. S-adenosyl methionine levels were markedly increased in pancreas of obese rats. Acute pancreatitis in obese rats led to glutathione oxidation and lower reduced glutathione levels in pancreas together with decreased activities of redox-sensitive phosphatases PP1, and PP2A. S-adenosyl methionine levels decreased but cystine levels increased markedly in pancreas upon pancreatitis. Acute pancreatitis triggered an increase in isoprostane levels in plasma and ascites in obese rats. Free fatty acid levels were extremely high in pancreatitis-associated ascitic fluid from obese rats and lipase was bound with great affinity to white adipose tissue, especially to areas of necrosis.

**Conclusions:**

Our results show that oxidative stress occurs locally and systemically in obese rats with pancreatitis favouring inactivation of protein phosphatases in pancreas, which would promote up-regulation of pro-inflammatory cytokines, and the increase of isoprostanes which might cause powerful pulmonary and renal vasoconstriction. Future studies are needed to confirm the translational relevance of the present findings obtained in a rat model of taurocholate-induced pancreatic damage and necrosis.

## Introduction

Acute pancreatitis is an initially localized inflammation of the pancreatic gland that frequently leads to local and systemic complications. The incidence of acute pancreatitis in the European Union and USA varies from 5 to 30 cases/100 000/year [1]. The overall mortality in patients with acute pancreatitis is around 5%, but this percentage increases up to 17–20% in patients with necrotizing pancreatitis due to multiple organ failure despite the therapeutic efforts established so far [2]. Obesity is a prognostic factor for severity in the evolution of acute pancreatitis since local and systemic complications are more frequent in obese patients than in non-obese ones [3]–[8]. Furthermore, patients with severe acute pancreatitis exhibit higher percentage of fat than those with mild acute pancreatitis [9]. In addition, obese Zucker rats exhibited TNF-α expression and higher mortality rate than controls in the experimental model of acute pancreatitis induced by taurocholate [10], [11].

Obesity is a pro-inflammatory condition [12]. Accordingly, obese subjects and animals exhibit high serum and tissue levels of pro-inflammatory cytokines, such as TNF-αand interleukin 6 [13]. The levels of pro-inflammatory interleukin IL-18 are also elevated in obese subjects, and simultaneous treatment with IL-12 and IL-18 causes severe acute pancreatitis in obese mice but only edematous pancreatitis in control mice [14]. A decrease in adiponectin levels is a feature of obese animals and it might contribute to the severity of pancreatitis since adiponectin exhibits anti-inflammatory properties and a deficiency in adiponectin causes severe pancreatitis in mice fed a high-fat diet, whereas its over-expression protects against tissue damage [15]. Adipose tissue and particularly the areas of fat necrosis are important sources of inflammatory mediators that may contribute to the systemic inflammatory response in acute pancreatitis [16]. Nevertheless, the mechanisms responsible for the increased severity of acute pancreatitis in obese subjects are still under investigation.

Obesity is associated with oxidative stress [17] and reactive oxygen species (ROS) are considered mediators of the inflammatory response and tissue damage in acute pancreatitis [18]. The involvement of oxidative stress in acute pancreatitis (AP) is evidenced by glutathione depletion and lipid peroxidation in the pancreas during experimental AP [19], [20]. Mice deficient in NADPH oxidase exhibited attenuation of cerulein-induced trypsin activation in the pancreas [21] and this ROS generating enzyme up-regulates IL-6 in cerulein-stimulated pancreatic AR42J acinar cells [22]. Xanthine oxidase triggers intracellular trypsinogen activation and zymogen granule damage in isolated pancreatic acini [23] and promotes leukocyte recruitment in the lung via induction of P-selectin [24], [25]. Moreover, high level of lipid peroxidation, measured as thiobarbituric acid reacting substances (TBARS), was found in pancreatitis-associated ascitic fluid during acute pancreatitis [26]. Taking into account all this background, the aim of the present work is to assess the role of oxidative stress and abdominal fat in the increased severity of acute pancreatitis in obese Zucker rats.

## Materials and Methods

### Animals

Male lean Zucker (control) rats (308±25 g b.w.) and obese Zucker rats (fa/fa) (433±34 g b.w.) were purchased from Harlan laboratories (Barcelona, Spain) and Charles River (Barcelona, Spain). Zucker rats are characterized by a mutation in the leptin receptor and show hyperphagia and other alterations similar to those that appear in human metabolic syndrome [27]–[29]. Lean Zucker rats were genetically identical to the obese Zucker rats used except for the leptin receptor mutation. They were fed on a standard laboratory diet and tap water *ad libitum* and were subjected to a 12 hour light-dark cycle. All animals received humane care according to the criteria outlined in the “*Guide for the Care and Use of Laboratory Animals*” prepared by the National Academy of Sciences and published by the National Institutes of Health (NIH publication 86-23 revised 1985). The Ethical Committee of the University of Valencia (Spain) approved the study protocol.

### Experimental model of acute pancreatitis

Induction and maintenance of anaesthesia was performed with isoflurane (Isoflo®) inhaled at 3%. The biliopancreatic duct was cannulated through the duodenum and the hepatic duct was closed by a small bulldog clamp. Acute necrotizing pancreatitis was induced by retrograde injection into the biliopancreatic duct of sodium taurocholate (3.5%) (Sigma) in a volume of 0.3 ml of 0.9% NaCl using an infusion pump (Harvard Instruments). Rats were sacrificed at 0, 1 and 6 h after the infusion of taurocholate, and were anaesthetized as previously mentioned prior to sacrifice. Plasma lipase activity was measured and histological studies were performed to confirm the appropriate induction of necrotizing pancreatitis.

### Study Design

In a first series of experiments, oxidative stress, abdominal fat and ascites were studied. Animals were distributed in the following groups: Lean rats at 0 h (n = 8), at 1 h after pancreatitis induction (n = 4), and at 6 h post-induction (n = 8); Obese rats at 0 h (n = 8), at 1 h post-induction (n = 4) and at 6 h post-induction (n = 10). In the second series of experiments, we assessed phosphatase activities in pancreas from lean rats at 0 h (n = 6) and at 6 h post-induction (n = 6) and in pancreas from obese rats at 0 h (n = 7) and at 6 h post-induction (n = 7). Consequently, 32 lean rats and 36 obese rats were used in total in the study.

### Assays

Standard curves and blank analysis without samples were used for all assays. All samples were run simultaneously in parallel, except for HPLC and mass spectrometry, where samples were run consecutively with standards at the beginning and at the end.

#### GSH

Reduced glutathione (GSH) levels were determined spectrophotometrically at 340 nm using glutathione-S-transferase and 1-chloro-2,4-dinitrobencene as in [20]. For GSH measurement, tissue samples were homogenized with 6% perchloric acid (PCA) containing 1 mM EDTA. Homogenates were centrifuged at 15,000 g for 15 minutes at 4°C. Acidic supernatants were used for these assays. Protein concentration was determined by the Pierce BCA Protein assay kit (Thermo, Rockford, USA).

#### Pancreatic lipase activity

Pancreatic lipase activity was determined in plasma, ascitic fluid, and white adipocyte tissue homogenate by the LIPASE-LQ kit (Spinreact, Girona, Spain).

#### Malondialdehyde measurement

Malondialdehyde (MDA) levels were determined in plasma and ascitic fluid according to Wong et al [30].

#### Isoprostane measurement

A representative free isoprostane, 15-isoprostane F_2t_, was determined in plasma and ascitic fluid by Enzyme Inmunoassay (Oxford Biomedical Research). Samples were purified by solid phase extraction using C_18_ Sep Pak (Waters) and determined following manufacturer instructions.

#### Triglyceride measurement

Plasma and ascitic fluid triglycerides were determined by the TRIGLYCERIDES-LQ kit (Spinreact, Girona, Spain).

#### Free fatty acid measurement

Plasma and ascitic fluid free fatty acids were determined by the Free Fatty Acid Quantification Kit (Biovision, California, USA).

#### GSSG and transulphuration pathway metabolites

The concentrations of GSSG, methionine, S-adenosyl methionine, cysteine and cystine were determined in the supernatants by high-performance liquid chromatography coupled to tandem mass spectrometry (HPLC-MS/MS) as follows.

Frozen tissue samples were homogenized in 400 µl of PBS and NEM 11 mM. PCA was then added to obtain a final concentration of 4% and centrifuged at 15,000 g for 15 min at 4°C. The concentrations of GSSG, methionine, S-adenosyl methionine, cysteine and cystine were determined in the supernatants by high-performance liquid chromatography coupled to tandem mass spectrometry (HPLC-MS/MS). The chromatographic system consisted of a Micromass QuatroTM triple-quadrupole mass spectrometer (Micromass, Manchester, UK) equipped with a Z-spray electrospray ionization source operating in the positive ion mode with a LC-10A Shimadzu (Shimadzu, Kyoto, Japan) coupled to the MassLynx software 4.1 for data acquisition and processing. Samples were analyzed by reversed-phase HPLC with a C18 Mediterranea SEA column (Teknokroma, Barcelona, Spain) (5.0×0.21 cm) with 3 µm particle size. In all cases, 20 µl of the supernatant were injected onto the analytical column. The mobile phase consisted of the following gradient system (min/%A/%B) (A, 0.5% formic acid; B, Isopropanol/Acetonitrile 50/50; 0,5% Formic Acid): 5/100/0, 10/0/100, 15/0/100, 15.10/100/0, and 60/100/0. The flow rate was set at 0.2 ml/min. Positive ion electrospray tandem mass spectra were recorded with the electrospray capillary set at 3 keV and a source block temperature of 120°C. Nitrogen was used as the drying and nebulizing gas at flow rates of 500 and 30 L/h, respectively. Argon at 1.5×10-3 mbar was used as the collision gas for collision-induced dissociation. An assay based on LC-MS/MS with multiple reaction monitoring was developed using the transitions m/z, cone energy (V), collision energy (eV) and retention time (min) for each compound that represents favorable fragmentation pathways for these protonated molecules. Calibration curves were obtained using six-point (0.01 to 100 µmol/l) standards (purchased from Sigma-Aldrich, Madrid, Spain) for each compound. The amounts of total metabolites were calculated based on the weight of the dissected striatum, and the results were expressed as nanomols per mg of protein.

#### Phosphatases: PP2A and PP1 activities

PP2A and PP1 activity were measured by Ser/Thr Phosphatase Assay System (Promega, Madison, USA) following manufacturer instructions with slight modifications. For PP2A activity, 10 mM tautomycetin was added to reaction buffer in order to inhibit PP1 [31].

### Tyrosine Phosphatase activity

Tyrosine Phosphatase activity was measured by Tyrosine Phosphatase Assay System (Promega, Madison, USA) following manufacturer instructions with slight modifications. Reaction buffer was 100 mM Tris-HCl (pH = 6.8), 50 mM DTT, 5 mM EDTA, 5 mM EGTA and 125 mM NaF as in [32] and Tyr-phosphopeptide 2 was used as substrate.

#### Western blotting

White adipocyte tissue specimens were frozen at −80°C until homogenization in extraction buffer (200 mg/ml) on ice. The extraction buffer contained 50 mM potassium phosphate buffer (pH 7.4), 0.1 mM EDTA, 0.5% Igepal, 30 mM sodium pyrophosphate, 50 mM sodium fluoride and 50 µM sodium orthovanadate. A protein inhibitor cocktail (Sigma) was added just before its use at a concentration of 4 µl/ml. The homogenates were sonicated with a Branson Sonicator SLPe for 30 sec (2 sec each pulse) at 30% of amplitude and centrifuged at 15,000 g for 15 min at 4°C.

Fifty micrograms of protein were separated in Criterion Gel 4–15% (Biorad) by electrophoresis and transferred to iBlot Gel transfer Stacks (nitrocellulose) (Invitrogen) membranes. Lipase and Erk 1/2 (p42/p44) were determined by western blotting and chemiluminiscence detection using the Phototope™-HRP Detection kit (Cell Signaling Technology). The following antibodies were used: antibody against pancreatic lipase (1/750) (Santa-Cruz) and antibody against phospho-p44/42 MAP kinase (Thr 202/Tyr 204) (1/1000) (Cell Signaling Technology).

### Statistical Analysis

Results are expressed as mean ± standard deviation (S.D.). Statistical analysis was performed in two steps. One-way analysis of variance (ANOVA) was performed first. When the overall comparison of groups was significant, differences between individual groups were investigated by the Scheffé test. Differences were considered to be significant at p<0.05.

## Results

### Glutathione depletion and oxidation during acute pancreatitis in lean and obese rats

**Figure 1:**
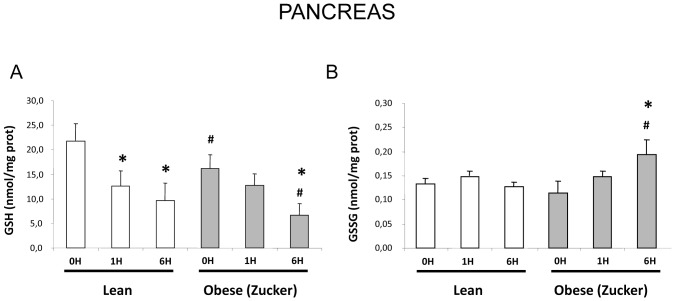
Pancreatic GSH and GSSG levels in lean and obese rats with acute pancreatitis. Pancreatic levels of GSH (A) and GSSG (B) in lean and obese (Zucker) rats at 0, 1 and 6 hours after induction of acute pancreatitis. The number of rats per group was 8 for lean rats sacrificed at 0 or 6 h and for obese rats sacrificed at 0 h, 10 for obese rats sacrificed at 6 h, and 4 for lean and obese rats sacrificed at 1 h. The statistical difference is indicated as follows: * P<0.05 vs. time “0”. **^#^** P<0.05 in obese vs. lean in the same conditions.

Figure 1 shows that basal reduced glutathione (GSH) levels in pancreas from obese rats were lower than in pancreas from lean rats. Upon induction of pancreatitis, glutathione depletion occurred in both strains, but at 6 h pancreatic GSH levels were significantly lower in obese than in lean rats. Oxidized glutathione (GSSG) levels did not change significantly during pancreatitis in lean rats, but they increased at 6 h in obese rats. Therefore, glutathione oxidation occurred in obese rats during pancreatitis evidenced by an increase in the GSSG/GSH)×1000 ratio [28.4**±**8.7 in obese rats vs 13.5±2.1 in lean rats at 6 h after induction of pancreatitis].

**Figure 2:**
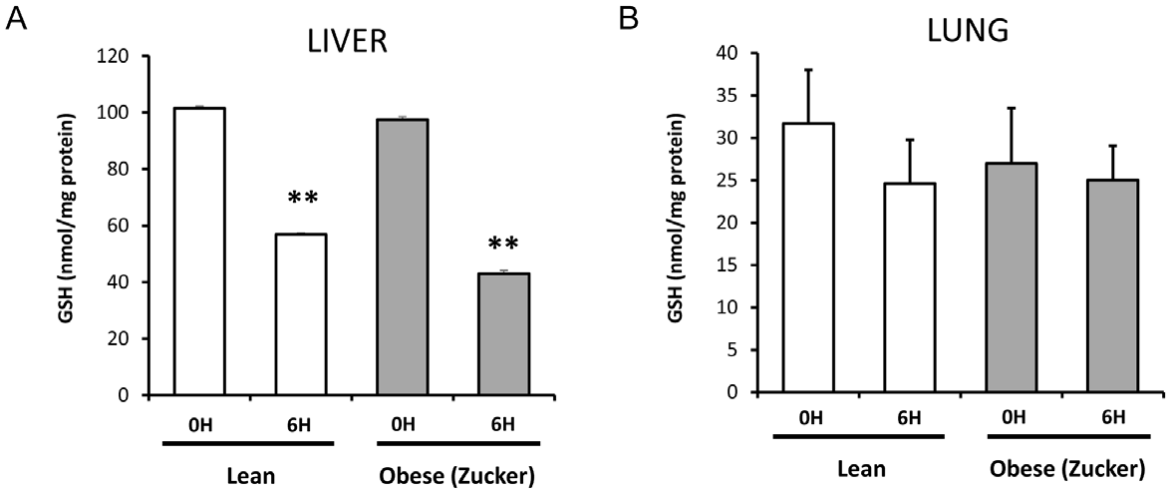
Hepatic and pulmonary GSH levels in lean and obese rats with acute pancreatitis. Levels of GSH in liver (A) and in lung (B) in lean and obese (Zucker) rats at 0 and 6 hours after induction of acute pancreatitis. The number of rats per group was 8. The statistical difference is indicated as follows: ** P<0.01 vs. time “0”.

Regarding extrapancreatic tissues, such as liver and lung, GSH levels were depleted upon induction of pancreatitis in both lean and obese rats in the liver (Fig. 2). GSH did not diminish significantly in the lung of lean rats at 6 h after induction of pancreatitis, nor in obese rats (Fig. 2).

### Changes in the transulfuration pathway during pancreatitis in lean and obese rats

**Figure 3:**
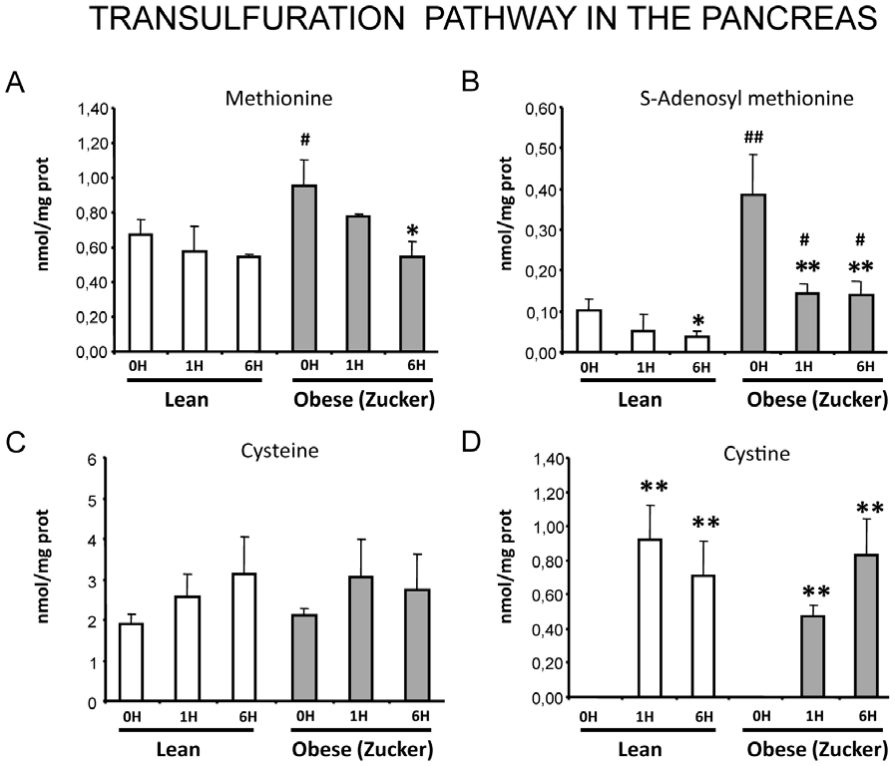
Characterization of the transulfuration pathway in the pancreas in acute pancreatitis. Levels of methionine (A), S-adenosyl methionine (B), cysteine (C) and cystine (D) in lean and obese (Zucker) rats at 0, 1 and 6 hours after induction of acute pancreatitis. The number of rats per group was 8 for lean rats sacrificed at 0 or 6 h and for obese rats sacrificed at 0 h, 10 for obese rats sacrificed at 6 h, and 4 for lean and obese rats sacrificed at 1 h. The statistical difference is indicated as follows: * P<0.05 and ** P<0.01 vs. time “0”. **^#^** P<0.05 and **^##^** P<0.01 in obese vs. lean in the same conditions.

Four key components of the transfulfuration pathway –methionine, S-adenosyl methionine, and the final products cyteine and cystine- were measured in pancreas during pancreatitis in lean and obese rats. Methionine and S-adenosyl methionine levels -especially the later- were higher in pancreas from obese rats than from lean rats in basal conditions, whereas cysteine levels were not significantly different between lean and obese rats (Fig. 3). It is worth noting that S-adenosyl methionine levels were 4-fold higher in pancreas from obese rats than in controls. Upon pancreatitis there was a marked decrease in S-adenosyl methionine levels, especially in obese rats (Fig. 3). Methionine levels only decreased during pancreatitis in obese rats. Although cysteine levels did not change significantly during pancreatitis, there was a remarkable increase in its oxidized form –cystine- at 1 and 6 h post-induction in both groups (Fig. 3).

### Loss of protein phosphatase activity during pancreatitis in lean and obese rats

**Figure 4:**
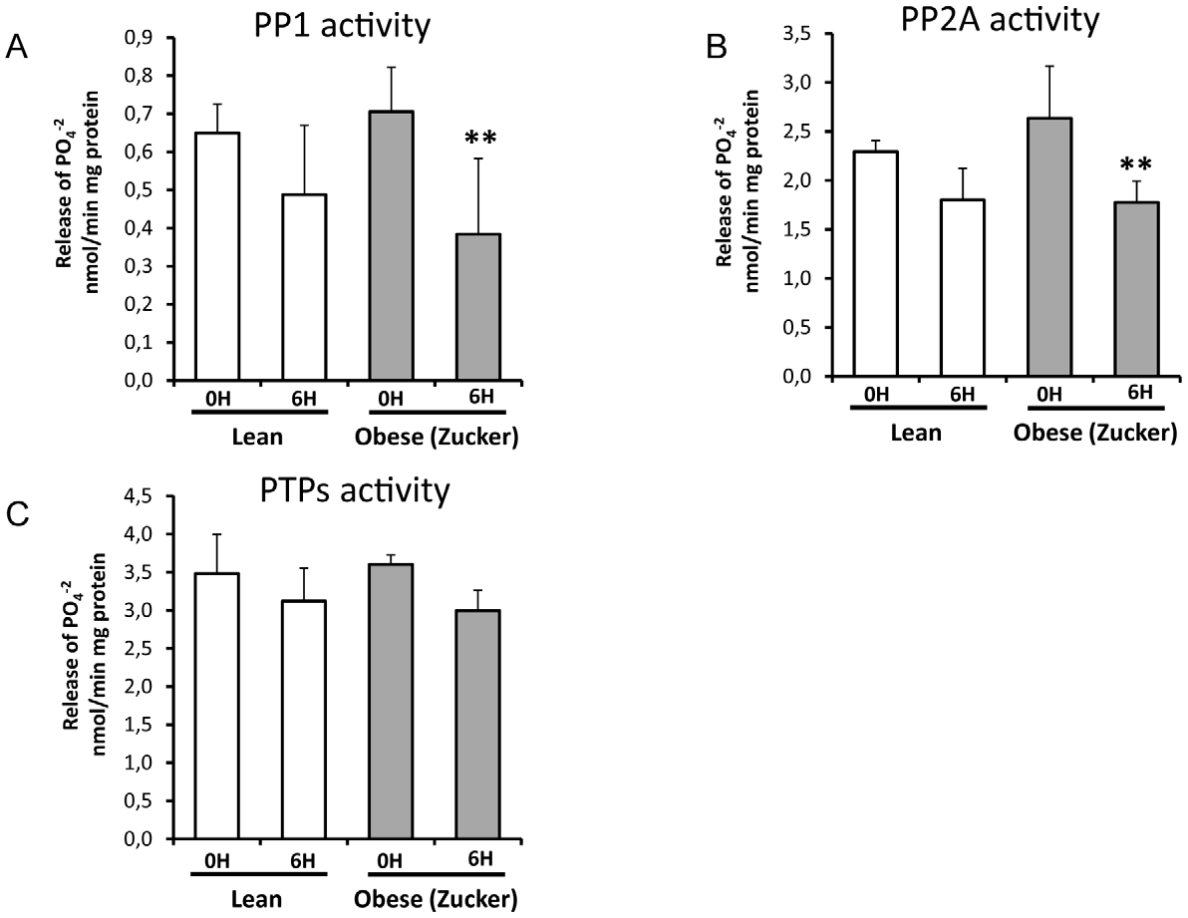
Phosphatase activity in lean and obese rats at 0 and 6 hours after induction of acute pancreatitis. Serine/threonine protein phosphatases activity -PP1 (A) and PP2A (B)- and tyrosin phosphatases (PTPs) activity (C) are shown as release of PO_4_^−2^ per min. Results are normalized by miligrams of protein. The number of rats per group was 6 for lean rats sacrificed at 0 or 6 h, and 7 for obese (Zucker) rats sacrificed at 0 h or 6 h. The statistical difference is indicated as follows: ** P<0.01 vs. time “0”.

Figure 4 shows that the activities of serine/threonine protein phosphatases PP1 and PP2A in pancreas decreased significantly at 6 h after induction of pancreatitis only in obese rats, but not in lean rats. Indeed, the percentage of reduction was 25% for PP1 and 21% for PP2A in lean rats versus 46% for PP1 and 32% for PP2A in obese rats. Regarding tyrosine phosphatase activities, they did not change significantly in lean rats nor in obese rats at 6 h after pancreatitis induction, although in obese rats the reduction in this phosphatase activity was around 17% upon pancreatitis.

### Malondialdehyde and isoprostane levels in pancreatitis in lean and obese rats

**Figure 5:**
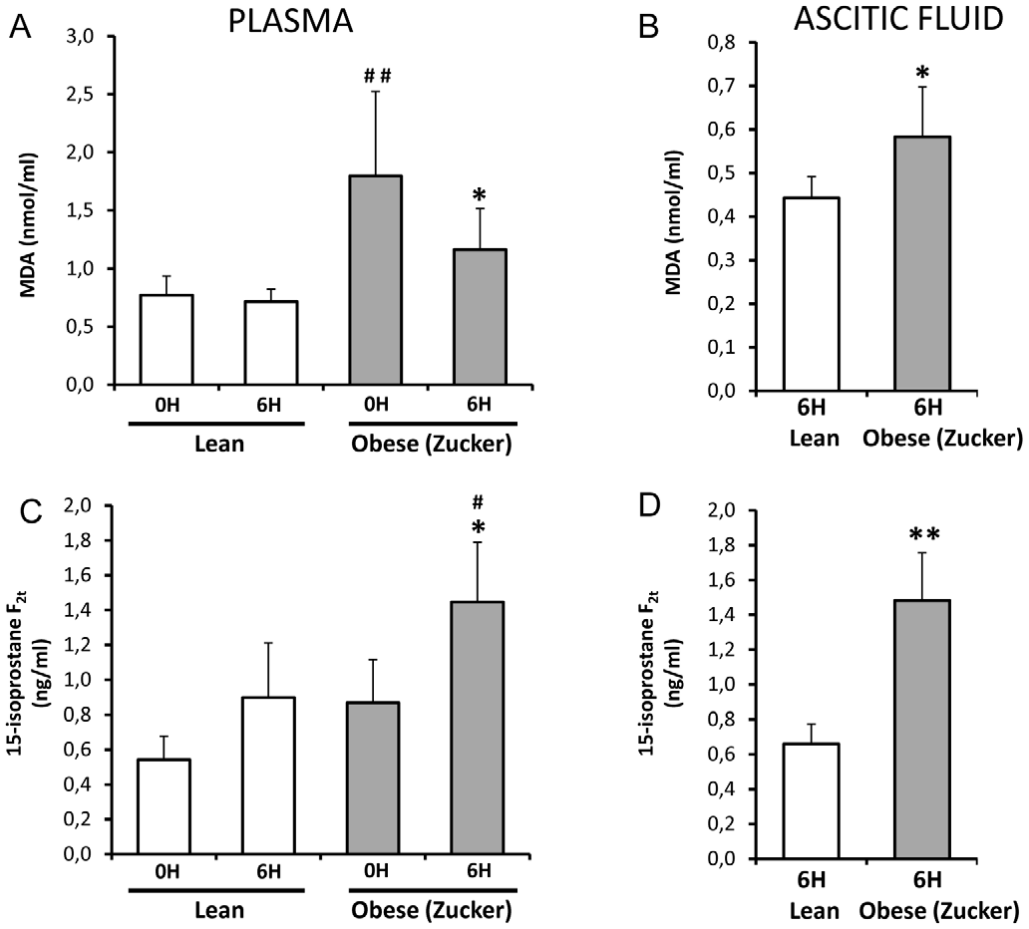
Lipid peroxidation in plasma and ascitic fluid from lean and obese rats with acute pancreatitis. Levels of MDA and 15-isoprostane F_2t_ in plasma (A,C) and ascitic fluid (B,D) in lean and obese (Zucker) rats at 0 and 6 hours after induction of acute pancreatitis. The number of rats per group was 8 for determinations in plasma from lean and obese rats at 0 or 6 h and in ascitic fluid from obese rats at 6 h; 4 for determinations in ascitic fluid from lean rats at 6 h. The statistical difference is indicated as follows: * P<0.05 and ** P<0.01 vs. time “0”. **^#^** P<0.05 and **^##^** P<0.01 obese vs. lean in the same conditions.

Malondialdehyde (MDA) and isoprostane levels were measured as biomarkers of lipid peroxidation *in vivo* [33]. MDA and isoprostane levels in plasma were higher in obese rats than in lean rats in basal conditions (Fig. 5A and C). Plasma MDA levels did not change significantly at 6 h after induction of pancreatitis in lean rats, whereas they diminished significantly at 6 h post-induction in obese rats (Fig. 5A). In contrast, isoprostane levels increased significantly during pancreatitis only in obese rats (Fig. 5C).

In ascites, MDA and isoprostane concentrations were always higher in obese than in lean rats (Fig. 5 B and D). Furthermore, the total amounts of MDA and isoprotanes in ascites, calculated taking into account the total ascites volume, were much higher in obese rats than in lean rats [4.6±2.7 nmols of MDA and 9.6±3.6 ng of isoprostanes in ascites from obese rats vs. 1.5±0.9 nmols of MDA and 1.09±0.7 ng of isoprostanes in ascites of lean rats].

MDA levels were also measured in areas of fat necrosis in rats with pancreatitis and they were remarkably higher when compared with levels in abdominal fat without necrosis (results not shown), in accordance with the high TBARS levels in pancreatitis-associated fat necrosis recently reported by Franco-Pons et al [16].

### Triglyceride and free fatty acid levels and lipase activity in plasma and ascites from lean and obese rats with pancreatitis

**Figure 6:**
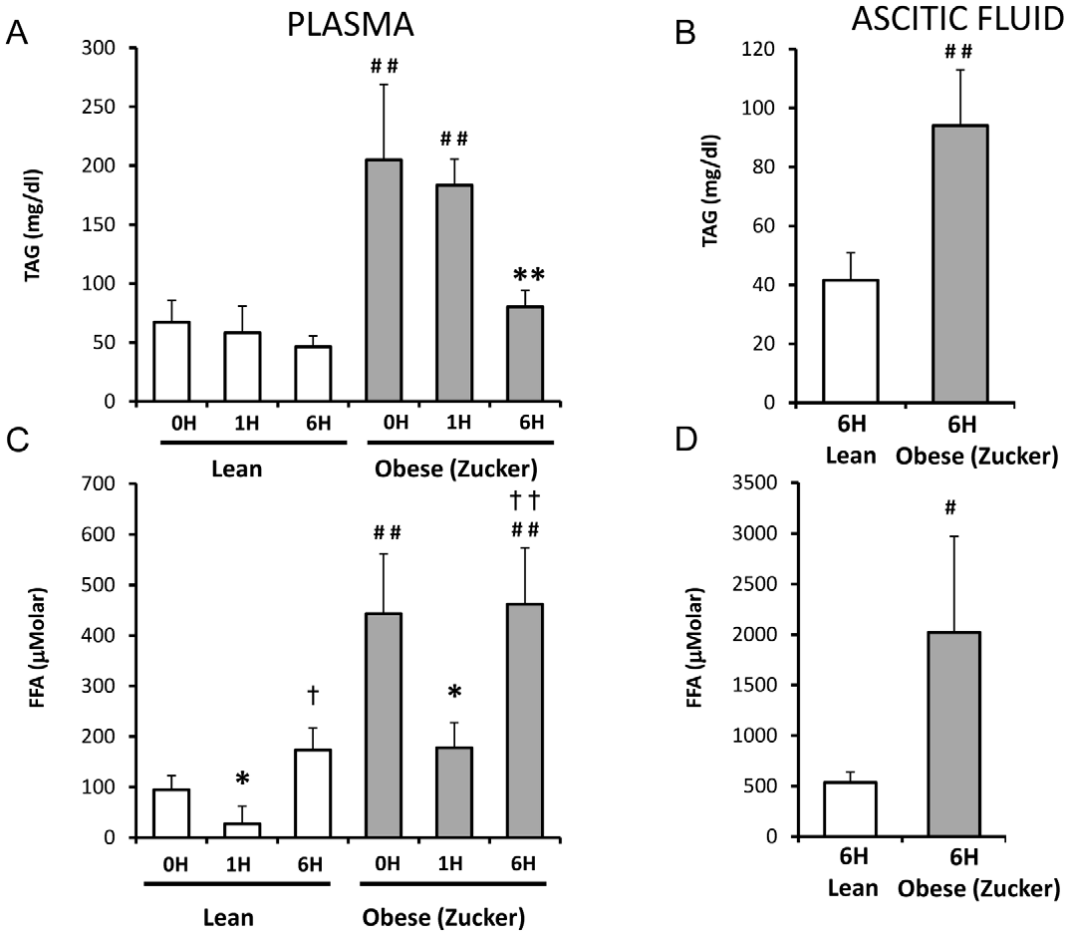
Lipid profile in plasma and ascitic fluid from lean and obese rats with acute pancreatitis. Concentration of triacylgricerides (TAG) and free fatty acids (FFA) in plasma (A,C) and ascitic fluid (B,D) in lean and obese (Zucker) rats at 0, 1 and 6 hours after induction of acute pancreatitis. The number of rats per group was 8 for determinations in plasma from lean and obese rats at 0 or 6 h and in ascitic fluid from obese rats at 6 h; 4 for determinations in plasma from lean and obese rats at 1 h and in ascitic fluid from lean rats at 6 h. The statistical difference is indicated as follows: * P<0.05 and ** P<0.01 vs. time “0”. **^#^** P<0.05 and **^##^** P<0.01 obese vs. lean in the same conditions. **^†^** P<0.05 and **^††^** P<0.01 obese 6 h vs. obese 1 h.

Triglyceride levels in plasma were more than 2-fold higher in obese rats than in lean rats in basal conditions (Fig. 6A). After induction of pancreatitis, triglyceride levels diminished significantly only in obese rats [60% decrease] (Fig. 6A). As expected, lipase activity increased markedly and progressively in lean and obese rats after induction of pancreatitis (results not shown). The profile of lipase activity in plasma was similar at 1 h, but at 6 h post-induction lipase activity was significantly higher in lean rats than in obese rats. Free fatty acid levels were more than 3-fold higher in plasma from obese rats than in lean rats in basal conditions (Fig. 6C). After induction of pancreatitis there is a transient decrease in free fatty acid levels (Fig. 6C). Indeed, at 1 h free fatty acid levels were reduced by 70% in lean rats and by 55% in obese rats. Later these levels increased rapidly and markedly as free fatty acid levels in lean rats were 6-fold higher at 6 h post-induction than at 1 h and almost 2-fold higher than basal levels, and in obese rats the values at 6 h were more than 2-fold higher than at 1 h (Fig. 6C). In any condition, free fatty acid levels were always remarkably higher in obese rats and in lean rats.

In the ascitic fluid, triglyceride levels were more than 2-fold higher in obese rats than in lean rats, and free fatty acid levels were dramatically higher –around 4-fold- in obese rats than in lean rats (6D and E). It should be highlighted that free fatty acid levels were tremendously high in ascites from obese rats –around 2000 µM-.

### Fat necrosis and lipase activity in abdominal fat in pancreatitis in lean and obese rats

**Figure 7:**
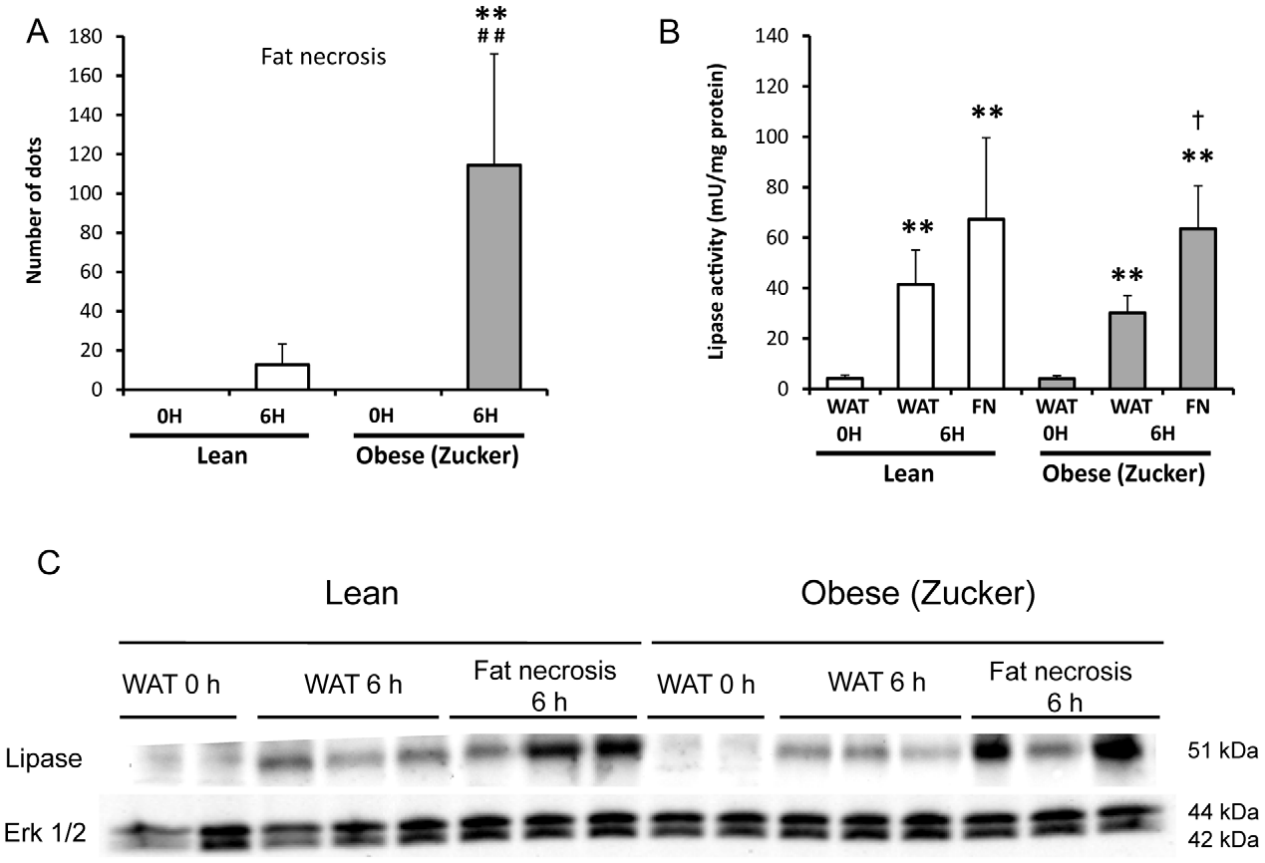
Fat necrosis and pancreatic lipase in abdominal white adipose tissue from lean and obese rats with acute pancreatitis. Macroscopic quantification of fat necrosis in lean and obese (Zucker) rats at 0 and 6 hours after induction of acute pancreatitis (A). Presence of pancratic lipase in adipose tissue in acute pancreatitis is illustrated by the increase of pancreatic lipase activity in white adipose tissue (WAT) and fat necrosis (FN) (B). A representative image of the presence of pancreatic lipase in WAT and FN is shown (C). Erk 1/2 was used as loading control. The number of rats per group was 8–10 for A and B, and 6–9 for C. The statistical difference is indicated as follows: ** P<0.01 vs. time “0”. **^##^** P<0.01 obese vs. lean in the same conditions. **^†^** P<0.05 fat necrosis vs. WAT at 6 hours in obese rats.

The amount of necrosis present in abdominal fat during pancreatitis was quantified by the number of dots of fat necrosis. Interestingly, the amount of fat necrosis was much lower in lean rats than in obese rats in which necrosis was observed to be around 10- fold higher (Fig. 7A). It is worth noting that lipase activity was very low in abdominal fat in both lean and obese rats under basal conditions, but after induction of pancreatitis lipase activity in fat increased markedly in both groups and this increase was even higher in areas of fat necrosis (Fig. 7B). The higher presence of lipase in fat after induction of pancreatitis was confirmed by western blotting (Fig. 7C), and occurred especially in areas of fat necrosis in obese rats with pancreatitis.

## Discussion

### Role of oxidative stress and isoprostanes in pancreatitis and obesity

Reactive oxygen species (ROS) at a high level are cytotoxic causing necrosis and at low level may serve as signalling messengers also contributing to the inflammatory process [34]–[36]. Indeed, pro-oxidant NADPH oxidase and xanthine oxidase activities are mediators of the inflammatory response and tissue damage in pancreatitis [21]–[23]. However, the role of ROS in pancreatitis may be controversial because they might be beneficial during acute pancreatitis by promoting apoptosis, since they mediate cerulein- or cholecystokinin-induced apoptosis in pancreatic acinar cells [37]. In addition, Ca^2+^-dependent ROS induced by bile acids trigger apoptosis in acinar cells protecting against necrosis [38]. In the present work we have studied the possible contribution of local and systemic oxidative stress to the increased severity of pancreatitis in obese animals.

Pancreatic glutathione depletion is a feature of acute pancreatitis and low basal levels of this major intracellular antioxidant are associated with increased mortality and tissue damage [20], [39], [40]. In the present work, we report that pancreatic GSH levels in basal conditions and at 6 h post-induction were lower in obese rats than in lean ones. Hence, this decrease in the antioxidant defense may itself contribute to increased pancreatic tissue damage in obese animals. We have also found that glutathione oxidation and consequently oxidative stress were present to a much higher extent in pancreas from obese than from lean rats. Furthermore, the decrease in the activities of redox-sensitive serine/threonine protein phosphatases PP1 and PP2A upon pancreatitis occurred in obese but not in lean animals. These protein phosphatases may be inactivated by oxidative stress [34], [36], [41]. The lower phosphatase activities found in pancreas from obese animals would lead to enhanced activation of MAP kinases and up-regulation of pro-inflammatory cytokines, explaining at least in part the higher levels of these cytokines in obese animals reported previously by Segersvard et al [10], [11]. Inactivation of tyrosin protein phosphatases –whose activity does not change significantly in the present work at 6 h post-induction- has been involved in the formation of pancreatic edema and in up-regulation of TNF-α [41], [42].

The transulfuration pathway was studied because it provides cysteine, whose levels are rate limiting in GSH synthesis. Hence, changes in the transulfuration pathway might contribute to pancreatic GSH depletion during pancreatitis and particularly to the lower GSH levels in the pancreas of obese rats. According to our results, the transulfuration pathway is not responsible for pancreatic GSH depletion in pancreatitis since cysteine levels did not decrease during pancreatitis. In contrast, pancreatic levels of cysteine and its oxidized form cystine increased markedly after induction of pancreatitis, probably as a result of GSH breakdown by pancreatic proteases. The remarkable increase in the cystine/cysteine ratio would favour oxidation of thiols in proteins and subsequently their inactivation. It is worth noting that pancreatitis in lean rats caused a remarkable increase in pancreatic cystine levels without changes in GSSG levels, indicating that intracellular cysteine oxidation occurred independently of changes in glutathione redox status. Hence, the pair cysteine/cystine may control the thiol redox status intracellularly in an independent manner from the pair GSH/GSSG.

Importantly, pancreatic levels of S-adenosyl methionine, one of the key metabolites of the transulfuration pathway, were markedly higher in basal conditions in obese animals and were depleted upon pancreatitis in both groups. The depletion of pancreatic S-adenosyl methionine levels in acute pancreatitis was already reported by Lu et al. [43] in pancreatitis induced by a choline-deficient ethionine-supplemented diet. The critical role of S-adenosyl methionine as a methyl donor might determine a specific regulation of gene expression through methylation of CpG islands or histones in pancreas related to a high dietary intake. Further research would be needed in this regard.

Concerning lipid peroxidation, the levels of both biomarkers MDA and isoprostanes were always higher in plasma and ascites of obese animals than in lean ones not only in basal conditions but also during pancreatitis. This finding would demonstrate a more intense systemic oxidative stress associated with pancreatitis in obese animals. Plasma lipid peroxides are associated with NF-κB activation and up-regulation of ICAM-1 in endothelial cells, leading to endothelial cell dysfunction and leukocyte cell adhesion [44]. Consequently, the increased lipid peroxidation would contribute to amplify the inflammatory response in obese animals. Unexpectedly, MDA levels were significantly lower in lean and obese rats at 6 h after induction of pancreatitis than in basal conditions. A possible explanation for this decrease could be the formation of adducts between MDA and proteins or the increased uptake of oxidized lipids by activated leukocytes.

Isoprostanes are endogenous bioactive prostaglandin-like compounds that are synthesized from esterified arachidonic acid by a free radical non-enzymatic reaction that occurs *in vivo* [45]. Hence, isoprostanes are reliable biomarkers of oxidative stress and have been involved in severe acute inflammation, such as septic shock [46]. In the present work, we have shown that basal levels of isoprostanes in plasma are higher in obese than in lean rats. Our results are in accordance with the higher plasma concentration of 8-epi-PGF_2α_ found in obese humans in comparison with non-obese ones [47], [48] as well as with the correlation between abdominal visceral and subcutaneous adipose tissue volumes and F_2_-isoprostane levels [49].

We have shown here that isoprostane levels are elevated upon acute pancreatitis in plasma and in the ascitic fluid, and this increase is especially marked in obese rats. Isoprostanes might be importantly involved in the pathophysiology of acute pancreatitis in obese subjects since they may activate thromboxane receptors causing powerful pulmonary and renal vasoconstriction [50]–[52]. It is noteworthy that lungs and kidneys are among the most affected extrapancreatic tissues in severe acute pancreatitis. In addition, isoprostanes induced vasoconstriction of human radial artery and contraction of vascular smooth muscle from porcine carotid artery via thromboxane A_2_ receptors [53], [54], and seem to be involved in angiotensin II-induced vasoconstriction in rabbit aorta or mesentery artery [55]. Furthermore, high isoprostane levels are considered an independent risk factor for coronary heart disease [33]. Consequently, high isoprostane levels might aggravate pancreatitis, a disease associated with profound changes in hemodynamic parameters [56], by causing powerful pulmonary and renal vasoconstriction. Administration of antioxidants may be considered in obese patients with acute pancreatitis to avoid the increase in isoprostane levels and the decrease in protein phosphatase activities.

### Pancreatitis-associated fat necrosis and obesity

Pancreatitis-associated ascitic fluid (PAAF) plays a key role in the pathogenesis of acute pancreatitis since it enhances the production of tumour necrosis factor α by pancreatic acinar cells [57], promotes macrophage activation [58], [26], and induces lung injury [59] and hepatocyte cell death [60]. Indeed, PAAF triggered activation of NF-κB and enhanced TNF-α production in peritoneal macrophages [58]. Furthermore, the lipid extract from ascitic fluid reduced the anti-inflammatory effect of 15-deoxy-PGJ2 as peroxisome proliferator-activated receptor gamma (PPARγ agonist [26].

Fat necrosis arises during pancreatitis in peripancreatic, mesenterial and retroperitoneal fat and is considered a consequence of the release of lipase and phospholipase from the pancreas into the peritoneal cavity [61], [1]. Recently, Closa and co-workers have reported that these areas of fat necrosis are an important source of pro-inflammatory cytokines, such as TNF-α, and exhibit less expression of anti-inflammatory interleukin 10 and contain an intense inflammatory infiltrate, particularly neutrophils [16]. Furthermore, peritoneal macrophages were strongly activated in the presence of necrotic white adipose tissue [16]. Therefore, abdominal fat necrosis may contribute to the systemic inflammatory response in acute pancreatitis [16]. In the present work, we have reported that the areas of pancreatitis-associated fat necrosis are much more abundant in obese rats than in lean ones. Therefore, the contribution of fat necrosis to the systemic inflammatory response would be more intense in obese animals. Our results show that lipase exhibits great affinity for binding to white adipose tissue in pancreatitis, especially in those areas of necrosis. Thus, lipase plays a key active role in the generation of fat necrosis particularly in obese animals. The lower lipase activity found in plasma of obese rats in comparison with lean rats at 6 h after pancreatitis might be due to higher binding of lipase to abdominal fat in obese animals.

Another consequence of the very high lipase activity in PAAF is the high concentration of free fatty acids in PAAF [62], which may explain the increase in free fatty acid in plasma upon pancreatitis that we report here. In the present work, we have also found a dramatic increase in free fatty acids in PAAF of obese rats in comparison with lean animals. Free fatty acids may trigger apoptosis of endothelial cells, cardiomyocytes, and pancreatic β-cells, and at high concentrations may be cytotoxic [63]–[65]. The dramatically high and potentially cytotoxic fatty acid levels found in PAAF from obese animals might affect permeability through the intestinal barrier favouring bacterial translocation. Although removal of PAAF was beneficial regarding survival rate in experimental acute pancreatitis [66], peritoneal lavage did not reduce mortality or morbility rates in clinical trials [67]. In this regard, it has been suggested that systemic inflammation was already triggered at the time of hospitalization and intervention [26].

We also report that plasma triglyceride levels are much higher in obese rats than in lean animals under basal conditions and during pancreatitis. Hypertriglyceridaemia is one of the important risk factors of acute pancreatitis [68] and recurrent pancreatitis is common in patients with severe hypertriglyceridaemia caused by mutations in lipoprotein lipase or apolipoprotein CII [69]. Accordingly, mice deficient in lipoprotein lipase exhibited severe hypertriglyceridaemia and enhanced susceptibility to acute pancreatitis [69]. Hence, high triglyceride levels may also contribute to the increased severity of pancreatitis in obese animals.

In conclusion, under basal conditions obese rats exhibited lower glutathione levels in the pancreas as well as higher isoprostane, triglyceride, and free fatty acid levels in plasma when compared with lean rats. S-adenosyl methionine levels were markedly increased in pancreas of obese rats. Necrotizing pancreatitis in obese rats was associated with more intense glutathione depletion and oxidation and decrease in protein phosphatase activities in the pancreas that would promote tissue damage and up-regulation of pro-inflammatory cytokines. S-adenosyl methionine decreased but cystine levels increased in pancreas upon pancreatitis favouring thiol oxidation of proteins. Acute pancreatitis triggered an increase of isoprostane levels in plasma and ascites, especially in obese rats. Free fatty acid levels were dramatically high in pancreatitis-associated ascitic fluid from obese rats parallel to an increase in binding of lipase to white adipose tissue, especially in areas of necrosis. Future studies are needed to confirm the translational relevance of the present findings obtained in a rat model of taurocholate-induced pancreatic damage and necrosis.
